# Identifying Key Factors for Implant Failure in a Periodontally Treated Population: A Retrospective Analysis

**DOI:** 10.4317/jced.63296

**Published:** 2025-10-01

**Authors:** Georgios S Chatzopoulos, Larry F Wolff

**Affiliations:** 1 DDS, MS, Department of Developmental and Surgical Sciences, Division of Periodontology, School of Dentistry, University of Minnesota, 515 Delaware Street SE, Minneapolis, MN,55455, USA & Department of Preventive Dentistry, Periodontology and Implant Biology, School of Dentistry, Aristotle University of Thessaloniki, 54124 Thessaloniki, Greece; 2 DDS, MS, PhD, Department of Developmental and Surgical Sciences, Division of Periodontology, School of Dentistry, University of Minnesota, 515 Delaware Street SE, Minneapolis, MN,55455, USA

## Abstract

**Background:**

The long-term success of dental implants is often compromised in patients with a history of periodontitis. This study aimed to identify the specific pre-implant clinical, demographic, and systemic risk factors associated with implant failure in a large cohort of periodontally susceptible patients who had received prior non-surgical therapy.

**Material and Methods:**

This retrospective cohort study utilized electronic health records from the multi-center BigMouth network (2011-2022). A final cohort of 434 patients with a history of periodontitis was analyzed at the patient level. Patients were stratified into an implant failure group (*n*=32) and a survival group (*n*=402). A comprehensive range of pre-implant variables, including detailed periodontal measurements from the most recent exam before surgery, demographics, and systemic conditions, was compared using t-tests and Chi-squared tests. Binary logistic regression was used to calculate odds ratios (OR) for significant predictors.

**Results:**

The analysis revealed that the severity of pre-existing periodontal disease was the primary predictor of failure. The failure group had a significantly higher pre-implant Mean Probing Depth (3.34 mm vs. 3.07 mm, *p*=0.0311) and more sites with PPD ≥ 4mm (*p*=0.0488). Logistic regression showed that for every 1 mm increase in Mean PPD, the odds of implant failure increased by a factor of 2.45 (OR=2.45, 95% CI: 1.08-5.56). Other patient-level factors, including demographics, systemic conditions, and medication use, as well as implant-level characteristics, were not significantly associated with implant failure.

**Conclusions:**

The severity of residual periodontal disease, specifically elevated pre-implant probing depths, is the most significant and powerful predictor of dental implant failure in patients with a history of non-surgically managed periodontitis. Achieving periodontal stability before surgery is paramount for long-term implant success in this high-risk population.

** Key words:**Dental Implants, Implant Failure, Periodontitis, Risk Factors, Probing Depth.

## Introduction

Dental implants have become a highly predicTable and successful treatment modality for the replacement of missing teeth, offering significant improvements in function, aesthetics, and quality of life [1,2]. However, the long-term success of implants can be compromised by patient-specific factors, with a history of chronic periodontitis being one of the most significant concerns [3]. Periodontitis is an inflammatory disease that leads to the destruction of the tooth-supporting apparatus, and the same bacterial pathogens and host-susceptibility factors that contribute to periodontitis can also lead to peri-implant diseases [4,5]. The 2023 guidelines from the European Federation of Periodontology (EFP) underscore the necessity of a consistent supportive care program to mitigate the risk of peri-implant diseases [6] Consequently, placing implants in patients with a history of periodontitis presents a unique clinical challenge, and a deeper understanding of the associated risks is essential for optimizing treatment outcomes.

There is a well-examined link between a patient’s periodontal history and the prognosis of their dental implants. High-level evidence from systematic reviews and meta-analyses repeatedly confirms that a background of periodontal disease increases the likelihood of implant loss, marginal bone resorption, and peri-implantitis [7-9]. The severity of the initial periodontal condition is also a key factor, with more advanced disease correlating with a greater number of implant complications [10]. A retrospective cohort study further illustrated this point by showing that implants replacing teeth lost to periodontitis were significantly more prone to early failure [11].

However, this negative association is not universally reported. Some research indicates that successful management of periodontal disease prior to implant placement can lead to survival rates comparable to those seen in periodontally healthy patients [12,13]. In agreement with this, another study found no significant difference in implant survival over both the short and long term when comparing individuals with and without chronic periodontitis [14]. Another retrospective study analyzed 322 implants in patients with a periodontitis diagnosis to evaluate whether the disease’s classification (extent, severity, or grade) predicted implant failure [15]. The results showed an overall implant failure rate of 5.6%, but found no statistically significant association between any of the 2017 periodontitis classifications and the implant treatment outcome.

While it is generally accepted that periodontally susceptible patients are at a higher risk for implant complications and failure, there is a critical need to identify specific, quantifiable pre-surgical factors that can effectively predict these adverse outcomes. The central question is whether implant failure in this population is primarily driven by systemic host-related factors—such as the severity of their underlying periodontal condition and other health issues—or by local, implant-specific characteristics like placement location and timing. Differentiating between these factors is crucial for developing targeted clinical protocols for risk assessment and patient management before, during, and after implant therapy.

This retrospective cohort study was designed to address this clinical question by analyzing a large dataset of patients with a history of chronic periodontitis who had undergone non-surgical periodontal therapy prior to receiving dental implants. The primary aim was to evaluate a comprehensive range of pre-implant variables, including detailed periodontal measurements (e.g., probing depths, bleeding on probing, mobility, furcation involvement), demographic characteristics, and systemic health conditions, to determine their association with implant failure. By focusing on clinical data from the most recent examination before implant placement, this study aimed to create a clear timeline for assessing the impact of baseline periodontal health on subsequent implant survival. To provide a thorough and statistically robust investigation, this study employed a dual analytical approach, examining the data at both the patient level and the implant level. This methodology allowed for the differentiation between patient-wide (systemic) risk factors and those specific to individual implants (local). The ultimate goal was to identify which pre-implant conditions, particularly the severity of residual periodontal disease, are most significantly associated with implant failure. The findings are intended to assist clinicians with evidence-based insights to better inform patient selection, treatment planning, and long-term maintenance strategies for placing dental implants in this high-risk and growing patient population.

It was hypothesized that the severity of residual periodontal disease at the time of implant placement is a significant predictor of implant failure. The primary aim of this retrospective cohort study was to identify the pre-implant clinical, demographic, and systemic risk factors associated with dental implant failure in patients with a history of chronic periodontitis who have been previously managed with non-surgical periodontal therapy.

## Material and Methods

This retrospective cohort study utilized patient data collected between 2011 and 2022 from the electronic health records of nine university dental clinics participating in the BigMouth network consortium [16]. The study protocol received a waiver of approval from the Institutional Review Board of the University of Minnesota (#STUDY00016865, approved 10/10/2022), and ethical clearance was granted by the BigMouth Consortium’s clinical review committee, with all research adhering to the principles of the Helsinki Declaration.

The study population was identified using the American Dental Association’s (ADA) standardized Current Dental Terminology (CDT) procedure codes. A final cohort of 434 patients was included, all of whom had a history of chronic periodontitis, had received non-surgical periodontal therapy, and subsequently underwent implant treatment, identified by the procedure code D6010 (surgical placement of an implant body).

Data for each patient was extracted and validated by independent analysts at The University of Texas Health Science Center at Houston. A comprehensive set of variables was collected from the electronic health records to assess potential risk factors. This included: demographic characteristics (age, gender, race, ethnicity); systemic health status (smoking, diabetes, hypertension, arthritis, osteoporosis); and medication use (antihypertensives, statins, antidepressants, thyroid medications, bisphosphonates, NSAIDs, immunosuppressants, corticosteroids). Most importantly, detailed pre-implant clinical periodontal data was extracted from the most recent examination prior to implant surgery, including Mean Probing Depth (PPD), the number of sites with PPD ≥ 4mm, Mean Clinical Attachment Loss (CAL), Bleeding on Probing Percentage (BOP%), highest recorded tooth mobility [17], and highest furcation involvement [18]. The number of missing teeth at this baseline exam and any subsequent tooth loss were also recorded.

- Statistical analysis

All statistical analyses were conducted to compare pre-implant characteristics between patients whose implants failed and those whose implants survived. A *p-value* of less than 0.05 was considered statistically significant. For continuous variables, such as age and clinical periodontal measurements, group means were compared using an independent samples t-test. For categorical data, including demographic variables, systemic conditions, and classifications of tooth mobility and furcation, proportional differences were assessed using the Chi-squared (χ²) test or Fisher’s Exact Test where appropriate for small sample sizes. Finally, a binary logistic regression was performed to calculate the Odds Ratio (OR) and 95% confidence interval for the most significant predictors, quantifying their association with implant failure. A secondary analysis was performed at the implant level. Chi-squared (χ²) tests were used to compare the frequency distributions of implant-specific categorical variables—including jaw location (maxilla vs. mandible), position (anterior vs. posterior), and placement timing (immediate vs. delayed)—between the two groups.

## Results

The analysis was conducted on a cohort of 434 patients with a history of periodontitis who had received non-surgical periodontal therapy. The comprehensive analysis of pre-implant risk factors is shown in  Table 1. To identify risk factors, these patients were stratified into two groups based on their implant outcomes: an “Implant Failure Group” consisting of 32 patients, and an “Implant Survival Group” comprising 402 patients. The baseline clinical, demographic, and systemic health characteristics recorded prior to implant placement were compared between these two groups.

The most notable differences were found in the pre-implant periodontal measurements. Patients in the implant failure group presented with a statistically significant higher mean probing depth (3.34 mm vs. 3.07 mm, *p*=0.0311) and a greater number of sites with probing depths of 4 mm or more. While not reaching statistical significance, this group also showed clear trends towards more severe disease across other clinical indicators, including a higher percentage of bleeding on probing (BOP %), greater mean clinical attachment loss (CAL), and a higher prevalence of advanced tooth mobility and furcation involvement (*p*>0.05). Furthermore, the average follow-up time was significantly shorter for the failure group (38.56 months) compared to the survival group (47.98 months), which is an expected consequence of early implant loss.

In contrast, the analysis of demographic and systemic factors revealed no significant differences between the groups. The average age, as well as the distributions for gender, race, and ethnicity, were comparable (*p*>0.05). Similarly, the prevalence of systemic conditions, including smoking, diabetes, hypertension, arthritis, and osteoporosis, did not differ significantly between the implant failure and survival groups (*p*>0.05). To investigate the potential influence of systemic health on implant outcomes, the use of several common medication classes was analyzed. The results indicated no statistically significant differences between the implant failure and survival groups.

The results of the logistic regression analysis were both statistically significant and clinically meaningful. The model revealed that for every 1 mm increase in a patient’s average pre-implant probing depth, the odds of their implant failing increased by a factor of 2.45 (OR = 2.45, 95% CI: 1.08 - 5.56, *p* = 0.0331). This model demonstrated that the mean pre-implant probing depth is a significant and powerful predictor of implant failure in this population of periodontitis patients. In addition, the logistic regression revealed that the number of pre-implant deep pockets (sites with PPD ≥ 4 mm) was a statistically significant predictor of implant failure (*p*=0.0488). The analysis yielded an odds ratio of 1.03 (OR: 1.03, 95% CI: 1.00-1.06, *p*=0.0488), indicating that for each additional site with a probing depth of 4 mm or greater, the odds of an implant failing increase by 3%. This finding further reinforces that the extent and severity of a patient’s pre-existing periodontal disease are critical determinants of the treatment outcome.

A secondary analysis was conducted at the implant level to determine if specific implant characteristics were associated with failure. The implant-level analysis of implant-specific variables is shown in  Table 2. The analysis included 1,228 implants, of which 32 failed and 1,196 survived. The results showed no statistically significant relationship (*p* > 0.05) between the evaluated implant-specific factors and their outcomes. Regarding arch location, implants in the maxilla constituted 59.4% of the failures and 54.1% of the survivals, showing no significant difference (*p*=0.5481). Similarly, the distribution between anterior and posterior positions was comparable, with posterior implants making up 78.1% of failures and 76.1% of survivals (*p*=0.7915). The timing of implant placement also showed no significant impact; delayed implants accounted for 75% of failures and 71.5% of survivals (*p*=0.6121). Ultimately, this analysis indicates that within this dataset, the specific arch location or placement protocol of an individual implant was not a significant factor in its survival.

Based on the significant findings of this study, a practical prognostic model was developed to help clinicians stratify risk of implant failure prior to implant placement in patients with a history of periodontitis is shown in  Table 3. This model categorizes patients into three distinct risk tiers—Low, Moderate, and High—based on their pre-implant mean PPD. A mean PPD of less than 3.0 mm is associated with a low risk and a favorable prognosis, while a mean PPD between 3.0 mm and 3.5 mm places a patient in a moderate risk category, where the odds of failure increase substantially and further non-surgical therapy is recommended. Patients presenting with a mean PPD greater than 3.5 mm fall into the high risk tier, for whom implant therapy is not advisable until their periodontal condition is significantly improved, potentially with surgical intervention. This simple, evidence-based tool uses the most powerful predictor identified in this analysis to provide clear, actionable guidance for treatment planning and patient communication.

## Discussion

This retrospective cohort study aimed to identify the key pre-implant risk factors associated with dental implant failure in a large, multi-center population of patients with a history of non-surgically managed periodontitis. The primary finding of this investigation is that the severity of residual periodontal disease at the time of implant placement is the most significant and powerful predictor of implant treatment outcomes. Specifically, a higher pre-implant mean probing depth (mean PPD) and a greater number of sites with PPD ≥ 4 mm were statistically significant predictors of future implant failure. The logistic regression analysis quantified this risk, revealing that for every 1 mm increase in a patient’s Mean PPD, the odds of implant failure increased by a factor of 2.45. In contrast, a wide range of other variables—including patient demographics, systemic health conditions, medication use, and even implant-specific characteristics like jaw location and placement timing—showed no significant association with implant failure. This strongly suggests that for this periodontally susceptible population, the success of implant therapy is dictated not by who the patient is or the specifics of the implant or implant placement, but by the clinical stability of their periodontal status before surgery.

These findings align with and add a crucial detail to the existing body of literature. Many systematic reviews and meta-analyses have consistently demonstrated that a history of periodontitis is a significant risk factor for implant failure and peri-implantitis [7,8,10]. Our study supports this overarching conclusion but refines it by identifying the specific clinical metrics that are most predictive. While some studies have suggested that implant outcomes in treated periodontitis patients can be comparable to those in healthy individuals [12,13], our results provide a potential explanation for these discrepancies. It is likely that the success of prior periodontal treatment, measured by the reduction of probing depths, is the key determinant. This would explain why a patient with a history of periodontitis but well-maintained, shallow probing depths (e.g., mean PPD < 3.0 mm) could achieve a successful outcome. Furthermore, our findings contrast with a recent study which found that the 2017 periodontitis classifications (Stage and Grade) were not predictive of implant failure [15]. Our results suggest that raw, quantitative clinical measurements like mean PPD may be more prognostically valuable for individual risk assessment than the broader classification categories.

While there is extensive evidence supporting the necessity of ongoing therapy to prevent the progression of periodontal disease, less data exists for the equivalent supportive care around dental implants [19,20]. However, a growing body of evidence indicates that regular supportive peri-implant therapy is essential for preventing and managing peri-implant diseases, particularly in patients with a history of periodontitis [21-25]. Studies show that patients who do not adhere to a consistent maintenance schedule exhibit a greater incidence of peri-implant bone loss, more plaque, and require more frequent surgical or antibiotic interventions to manage a complication [21-25]. The findings of the present study strongly reinforce the importance of a healthy periodontal state, demonstrating that the most significant predictors of implant failure were the deeper probing depths present before the implant was ever placed. This underscores that while ongoing supportive therapy is critical for long-term maintenance, achieving a state of periodontal stability prior to surgery is the foundational step in determining the ultimate success of the implant. The frequency of periodontal maintenance appointments per year did not show a significant association with the implant treatment outcome. The analysis revealed that the average number of periodontal maintenance (D4910) visits per year was comparable between the implant failure group (0.89 visits/year) and the implant survival group (0.98 visits/year), with a *p-value* of 0.6014. This indicates that within this cohort, the number of follow-up maintenance visits was not a differentiating factor for implant survival.

The primary strength of this study lies in its large, multi-center cohort drawn from the BigMouth network, which enhances the generalizability of the findings. Furthermore, the critical decision to use clinical data from the most recent exam prior to implant placement establishes a clear temporal relationship for risk assessment. However, the study is not without limitations. Its retrospective nature means it is reliant on the accuracy and completeness of electronic health records, where data on confounding variables like bone quality, occlusal factors (e.g., bruxism), and patient compliance may be unavailable. The definition of failure was limited to implant removal (CDT code D6100), which may not capture all instances of biological complications where a failing implant remains in situ. Furthermore, it should be noted that while a comprehensive literature review was conducted for context, this study was designed as a retrospective cohort analysis and not a systematic review; therefore, a formal systematic search protocol was not part of the methodology.

The findings of this study have significant clinical implications and point toward clear future research directions. Clinically, this work underscores the absolute necessity of achieving periodontal stability before proceeding with implant therapy. The proposed prognostic model, which stratifies patients into low, moderate, and high-risk tiers based on their pre-implant Mean PPD, can serve as a simple, evidence-based chairside tool to guide implant treatment planning and patient communication. This allows practitioners to translate an abstract risk into a tangible metric, making it easier to explain to a patient why they fall into a ‘moderate’ or ‘high’ risk category. Using this data-driven approach can enhance the informed consent process and help manage patient expectations regarding treatment timelines and the prognosis of implant therapy. For future research, prospective studies are needed to validate this prognostic model and to control for the unmeasured variables that were limitations in this retrospective analysis. Investigating the potential modifying effects of different periodontal therapy modalities (surgical vs. non-surgical) and the long-term impact of post-implant supportive care on these risk groups would be valuable next steps. Ultimately, this study reinforces a fundamental clinical principle: the long-term success of a dental implant is built upon the overall health status of the periodontal foundation that supports it.

## Conclusions

Within the limitations of this large, multi-center retrospective cohort study, the findings demonstrate that the primary determinant of dental implant failure in patients with a history of non-surgically managed periodontitis is the severity of their residual periodontal disease prior to surgery. Specifically, an elevated pre-implant mean probing depth and a greater number of deep pockets were statistically significant and powerful predictors of implant failure, while patient-level demographic and systemic factors, as well as implant-specific characteristics, were not. This study underscores the critical importance of achieving a stable and healthy periodontal foundation before proceeding with implant therapy. The proposed prognostic model, based on these findings, can serve as a valuable and practical chairside tool. It allows clinicians to stratify risk, guide evidence-based treatment planning, and facilitate clearer communication with patients, ultimately aiming to improve long-term implant outcomes in this high-risk population.

## Figures and Tables

**Table 1:** Comprehensive analysis of pre-implant risk factors.

Parameter	Implant Failure Group (n=32)	Implant Survival Group (n=402)	Total Population (n=434)	P-value*
Clinical Variables (Pre-Implant)				
BOP %	35.79%	29.77%	30.12%	0.1770
Sites with PPD >= 4mm	32.81	24.50	25.04	0.0488
Mean PPD (mm)	3.34	3.07	03.09	0.0311
Mean CAL (mm)	4.19	3.77	3.80	0.0805
Highest Mobility				
Class 0	28.12% (9)	40.05% (161)	39.35% (170)	
Class 1	40.62% (13)	35.07% (141)	35.48% (154)	
Class 2	25.00% (8)	19.90% (80)	20.28% (88)	
Class 3	6.25% (2)	4.98% (20)	5.07% (22)	0.1981
Highest Furcation				
Class 0	34.38% (11)	48.01% (193)	47.12% (204)	
Class 1	31.25% (10)	27.86% (112)	28.08% (122)	
Class 2	21.88% (7)	16.92% (68)	17.28% (75)	
Class 3	12.50% (4)	7.21% (29)	7.62% (33)	1.634
Missing Teeth	9.84	8.49	8.58	0.3524
Tooth Loss during Periodontal maintenance	1.88	1.34	1.37	0.2220
Periodontal maintenance visits/Year	0.89	0.98	0.97	0.6014
Follow-up Time (Months)	38.56	47.98	47.23	0.0245
Demographics				
Age	64.16	62.51	62.64	0.4207
Gender				0.4988
Female	59.38% (19)	52.99% (213)	53.46% (232)	
Male	40.62% (13)	47.01% (189)	46.54% (202)	
Race				0.8016
White	68.75% (22)	71.14% (286)	70.97% (308)	
Black or African American	15.62% (5)	14.93% (60)	14.98% (65)	
Asian	9.38% (3)	8.46% (34)	8.53% (37)	
Other/Unknown	6.25% (2)	5.47% (22)	5.53% (24)	
Ethnicity				0.4214
Not Hispanic or Latino	90.62% (29)	94.78% (381)	94.47% (410)	
Hispanic or Latino	9.38% (3)	5.22% (21)	5.53% (24)	
Systemic Conditions and Medications				
Smoker (% Yes)	12.50% (4)	13.18% (53)	13.13% (57)	1.0000
Diabetes (% Yes)	21.88% (7)	18.16% (73)	18.43% (80)	0.6385
Hypertension (% Yes)	53.12% (17)	46.52% (187)	46.99% (204)	0.4851
Arthritis (% Yes)	28.12% (9)	24.63% (99)	24.88% (108)	0.6628
Osteoporosis (% Yes)	9.38% (3)	11.44% (46)	11.31% (49)	0.7788
Antihypertensives (% Yes)	50.00% (16)	45.02% (181)	45.39% (197)	0.5983
Statins (% Yes)	37.50% (12)	39.55% (159)	39.40% (171)	0.8174
Antidepressants (% Yes)	18.75% (6)	16.42% (66)	16.59% (72)	0.7388
Thyroid Medication (% Yes)	15.62% (5)	17.66% (71)	17.51% (76)	0.7725
Bisphosphonates (% Yes)	6.25% (2)	4.98% (20)	5.07% (22)	0.7356
NSAIDs (% Yes)	46.88% (15)	48.01% (193)	47.93% (208)	0.8980
Immunosuppressants (% Yes)	3.12% (1)	4.48% (18)	4.38% (19)	0.1200
Corticosteroids (% Yes)	6.25% (2)	5.47% (22)	5.53% (24)	0.8407

* Bold Indicates a statistically significant result.

**Table 2:** Implant-level analysis of implant-specific variables.

Implant-Level Variable	Implant Failure Group (n=32)	Implant Survival Group (n=1,196)	P-value*
Location: Arch			0.5481
Maxilla	59.38% (19)	54.10% (647)	
Mandible	40.62% (13)	45.90% (549)	
Location: Position			0.7915
Anterior	21.88% (7)	23.91% (286)	
Posterior	78.12% (25)	76.09% (910)	
Timing of Placement			0.6121
Immediate	25.00% (8)	28.51% (341)	
Delayed	75.00% (24)	71.49% (855)	

* statistical significance determined at *p* <= 0.05

**Table 3:** Prognostic model for stratifying risk of implant failure prior to implant placement in patients with a history of periodontitis and treatment.

Risk Tier	Pre-Implant Mean PPD	Associated Risk & Interpretation	Clinical Consideration
Low Risk	< 3.0 mm	Patients in this tier have a periodontal status that is closer to the successfully treated group. The odds of implant failure are at their lowest for this cohort.	Proceed with implant therapy with a favorable prognosis. Continue with standard periodontal maintenance post-placement.
Moderate Risk	3.0 mm - 3.5 mm	This tier represents a significant warning. As Mean PPD increases, the risk of implant failure rises substantially. Our analysis showed an Odds Ratio of 2.45, meaning the odds of failure more than double for every 1mm increase in Mean PPD. Patients in this tier are approaching the average PPD seen in the failure group.	Implant therapy can be considered, but only after additional non-surgical therapy to reduce probing depths further. The patient must be informed of the elevated risk. A more rigorous post-implant maintenance schedule is highly recommended.
High Risk	> 3.5 mm	Patients in this tier have a pre-implant periodontal condition that is highly indicative of future complications. The odds of implant failure are substantially elevated.	Implant therapy is not recommended until the patient's periodontal condition is stabilized through more intensive treatment, which may include surgical therapy. Proceeding without improving these clinical parameters carries a very high risk of implant loss.

This model categorizes patients with a history of non-surgically treated periodontitis into three risk tiers based on their pre-implant mean probing pocket depth (PPD) - six surfaces of each dentition tooth (all teeth).

## Data Availability

The datasets used and/or analyzed during the current study are available from the corresponding author.
